# Dengue: an escalating public health problem in Latin America

**DOI:** 10.1179/2046904712Z.00000000046

**Published:** 2012-05

**Authors:** Roberto Tapia-Conyer, Miguel Betancourt-Cravioto, Jorge Méndez-Galván

**Affiliations:** 1 Carlos Slim Health Institute; 2 Hospital Infantil de México Federico Gómez, Ministry of Health, México City, México

**Keywords:** Dengue, Latin America, Urbanisation, Vector management, Vaccine

## Abstract

Dengue infection is a significant and escalating public health problem in Latin America. Its re-emergence and subsequent rise in the region over the past 50 years has largely been caused by a combination of a lack of political will, the radical growth of urban populations, migration flow and insufficient financial resources. Its increased incidence has been compounded by climate change, poor sanitation and extreme poverty, which lead to more breeding sites of the mosquito vector *Aedes aegypti*. In order to control dengue effectively, an integrated approach incorporating vector management and environmental and social solutions is required. To achieve success, these programmes require commitment and responses at both national and community level. The development of a vaccine is a vital tool in the fight against dengue. For successful introduction, those implementing vaccination need to be educated on the value of such a strategy. Effective political leadership, innovative financial mechanisms and co-operation across all disciplines, sectors and national borders are essential to eradication of the disease.

## Introduction

With more than 50 million cases reported to the World Health Organization (WHO) each year, dengue is now regarded as the world’s most important mosquito-borne viral disease.^1^ However, 60% of these reports are from the Americas, predominantly Latin America where the disease has re-emerged owing to re-infestation by the dengue vector *Aedes aegypti* following a period of eradication.^2^

The pattern of eradication of *A. aegypti*, its re-infestation and subsequent re-emergence of dengue in Latin America may serve as a warning of the challenges faced by other dengue-endemic regions such as South-east Asia, which has the highest dengue mortality.^2^ Clinical, political and socio-economic factors have contributed to the re-emergence of the disease and integrated responses are required at national, regional and global levels. To bring about these changes, however, there will need to be significant changes in social and political attitudes.

This article discusses the increasing impact of dengue in Latin America.

## The Re-Emergence of Dengue in Latin America

Following the last recorded outbreak of dengue in continental USA, in 1945 in the Mississippi Delta, the Pan American Health Organization (PAHO) recommended an *A. aegypti* eradication programme in 1947.^3^ This resulted in the Americas being an almost dengue-free zone from 1952 to 1965, when 19 Latin and Central American countries were certified as being free of *A. aegypti*. However, following an interruption in the vector control campaign, some countries in the region were re-infested with *A. aegypti* in 1967 and the first reports of dengue fever occurred soon after in 1968.^3^

## The Spread of Dengue in Latin America

**Figure 1:**
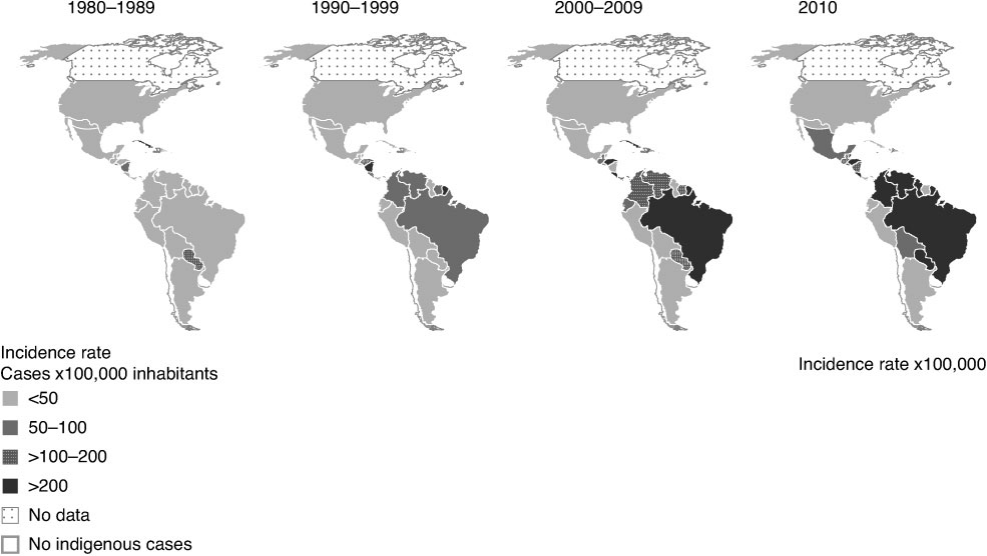
Average dengue incidence per 100,000 by country in the Americas, 1980–2010^4^

Since its re-emergence in Latin America, dengue has spread dramatically throughout the region (Fig. 1).^4^ The number of dengue cases has risen from 1,033,417 in the 1980s to 2,725,405 in the 1990s, and 4,759,007 between 2000 and 2007.^5^ Between 2001 and 2009, six countries accounted for more than 75% of all cases in the region: Venezuela, Brazil, Costa Rica, Colombia, Honduras and Mexico.^2^ Furthermore, a change in the age-group profile of the disease was identified in the 2007 epidemic in Brazil.^6^ Children were increasingly affected with severe dengue, more closely resembling the epidemiological profile in South-east Asia.

## The Current Situation in Latin America

Table 1 shows the number of cases of dengue fever and severe dengue as well as the incidence rate of dengue fever, the number of deaths and the case–fatality rate for 2010 (PAHO epidemiological week 52).^7^

**Table 1:** Cases of dengue and severe dengue in the Americas: incidence rate of dengue fever, number of deaths and case–fatality rate for 2010 (epidemiological week 52)^7^

Americas sub-region	Dengue fever	Incidence rate/100,000	Severe dengue	Deaths	Case–fatality rate
North & Central America & Mexico	205,756	140·0	10,411	152	1·46
Andean	305,744	294·1	19,744	224	1·13
Southern zone	1,019,130	418·9	16,570	688	4·15
Hispanic Caribbean	32,817	138·5	1,058	84	7·94
Caribbean	99,829	1258·1	1,171	46	3·93
**Total**	**1,663,276**	**316·3**	**48,954**	**1194**	**2·44**

By epidemiological week 50 in 2011, over 1 million cases of dengue fever had been reported throughout most Latin American countries, with over 18,000 cases of severe dengue and 716 deaths.^8^ All four dengue serotypes are circulating in the region.^9^

## Factors Influencing Re-Infestation and the Challenges to Containment

Various political, environmental and social factors influenced re-emergence of the disease in the region in the late 1960s.^10^ Following the passing of PAHO’s resolution on vector control in 1947, only some Latin American countries had the political will to work on eradicating the vector.^10^ Even in countries that successfully eliminated *A. aegypti*, interest in maintaining those efforts decreased over time. Furthermore, a slow response to re-infestation together with limited national budgets for vector control exacerbated the situation.^10^

**Figure 2:**
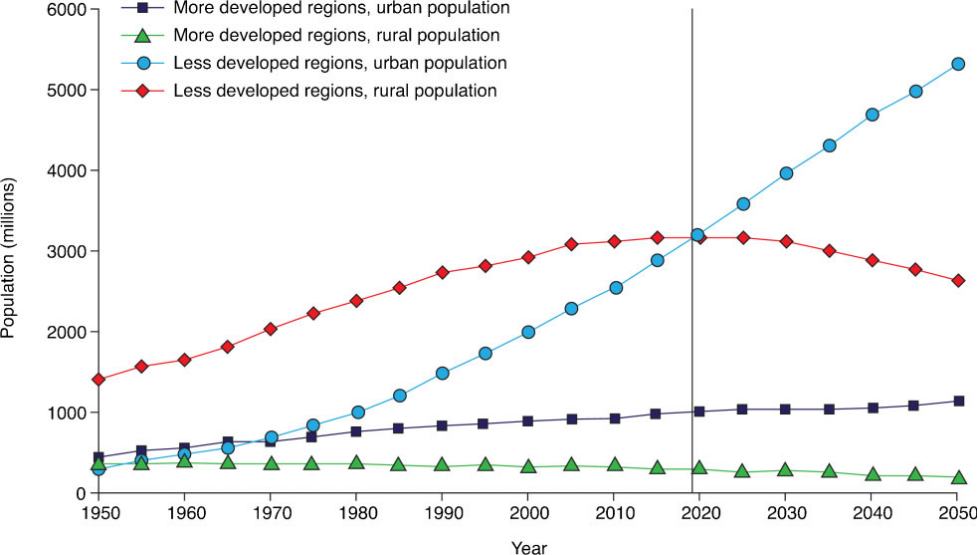
Global urban and rural population growth in developed and underdeveloped areas from 1950 to 2050.^11^ Reprinted from Lancet Infect Dis, 11, Alirol E, Getaz L, Stoll B, Chappuis F, Loutan L, Urbanisation and infectious diseases in a globalised world, 131–241, Copyright 2012, with permission from Elsevier

Other determinants of the re-emergence of dengue have played a continuing role in its further dissemination. Dengue is largely an urban disease and, in the Americas, international travel and large-scale migration across the continent and changes from a rural to an urban environment have resulted in unprecedented urbanisation and the formation of mega-cities.10,^11^ The accompanying overcrowding, poor sanitation and extreme poverty are optimal conditions for the establishment of vector breeding sites and dengue epidemics.^12^ The impact of urbanisation is particularly significant in less developed countries such as those in Latin America where the local infrastructure is minimal and they are less able to cope with a swelling population.^11^ Epidemics may become even more widespread in underdeveloped regions where the urban population is predicted to double by 2050 (Fig. 2).^11^

Although the link between climate change and dengue transmission is controversial, the intergovernmental panel on climate change and PAHO have concluded that climate change, leading to heavy rainfall, high temperatures and even drought, would cause a rise in infectious diseases such as dengue.^13^ In fact, a 2008 study in Mexico found an association in many states between the incidence of dengue and increased temperatures and rainfall.^13^

Another consequence of uncontrolled urbanisation and climate change is an inadequate water supply, leading to the domestic practice of storing water in containers, which serve as ideal vector breeding sites.10,^13^ The abundance of these and other containers capable of holding water has led to increased vector densities and virus transmission.

## Approaches to the Containment of Dengue

Control of dengue in the region will require an integrated approach to eradicate *A. aegypti* and improve living conditions and sanitation and, to be successful, national and community commitment will be needed.

PAHO has adopted a strategy to combat dengue through the destruction of *A. aegypti* using pesticides or disruption or removal of its habitat, known as the integrated management strategy for dengue prevention and control (IMS-Dengue).^14^ The initiative aims to promote the integration of key components for dengue prevention and control, including integrated vector management (IVM). To sustain the benefits of vector control, the programmes emphasise collaboration between local communities and community leaders through social mobilisation and communication, as well as an interdisciplinary approach. IMS-Dengue and IVM will be discussed in further detail in this supplement in the article ‘Community participation in the prevention and control of dengue: the *patio limpio* strategy in Mexico’.^15^

Government and community participation is particularly necessary following natural disasters such as floods. Strong and effective disease prevention and control measures are required before, during and, most importantly, after these events when public awareness of interventions is decreasing.

Reduction of vector breeding sites, such as used tyres, seems to be the most effective way to control the disease.^12^ With increasing numbers of mosquito breeding sites, a basic cultural and societal shift in attitude to the storage of water and sanitation is required, which might be addressed by programmes such as IMS-Dengue.

Control of dengue in the region relies predominantly on funds being assigned for this purpose. Such funds, however, are rarely sufficient for the financial and health burden that dengue imposes. Recognition of the economic impact of dengue at both regional and national levels, coupled with scientific and social awareness, might facilitate appropriate apportioning of financial resources.^16^

In addition to a vaccine, the introduction of cost-effective technologies to impede vector breeding will be essential. To this end, effective leadership and commitment to act systematically using a multidisciplinary and inter-sectoral approach, rather than discrete entities, are likely to have a significant impact on the disease.^12^

## Preparation for Introduction of a Dengue Vaccine

With the lead candidate vaccine against dengue currently in the advanced stages of clinical development,17 the availability of a licensed dengue vaccine is increasingly likely. If it is to be successfully introduced, however, several factors need to be considered.

These include the need for effective disease surveillance systems and laboratory networks that enable the determination of benchmark indicators for measuring the effects of the vaccine. The age-groups at risk of the disease, based on serological profiles, must be defined, as well as the vaccination strategies and target regions for use of the vaccine.^16^

In addition, local regulatory requirements must be anticipated by each country in the region to ensure that the vaccine is incorporated into national immunisation programmes as soon as it becomes available. Simultaneously, *ad hoc* decision-making bodies may be required as any delays in vaccination will inevitably lead to loss of life from the disease. Specially designed training programmes on the need for vaccination and its role in ongoing vector control strategies should therefore be made available to all those involved in its implementation.

Innovative mechanisms should be adopted to finance vaccination programmes, including cross-border schemes, to ensure that access to these programmes is not restricted for an individual nation because of inadequate resources.^16^

Finally, it is also essential to continue a vector control strategy, even with the availability of a dengue vaccine to limit the transmission of other infectious diseases for which *A. aegypti* is a vector.

## Conclusion

The substantial burden imposed by dengue in Latin America encompasses all aspects of society and an effective prevention and control response must be widespread and inter-sectoral. Public policies that effectively influence the determinants of disease transmission, with the vector at the centre of the strategy, should be promoted and strengthened in order to have the greatest impact on eliminating the disease.

Although an effective dengue vaccination programme is vital, to be truly successful it must be accompanied by permanent changes in political and social attitudes to dengue. Therefore, effective leadership and commitment is paramount to contain and eventually eradicate dengue.

## References

[1] Guzman MG, Halstead SB, Artsob H, Buchy P, Farrar J, Gubler DJ (2010). Dengue: a continuing global threat.. Nat Rev Microbiol..

[2] World Health Organization (2010). Working to Overcome the Global Impact of Neglected Tropical Diseases. First WHO Report on Neglected Tropical Diseases.

[3] Pan American Health Organization A timeline for dengue in the Americas to December 31, 2000 and noted first occurences. June 2001. [cited 18 December 2011].. http://www.paho.org/english/hcp/hct/vbd/dengue_history.htm.

[4] Pan American Health Organization Epidemiological alert: Update on Dengue Situation in the Americas. [updated 17 Februrary 2011; cited 18 December 2011]. Available from:. http://new.paho.org/hq/dmdocuments/2011/EPI-Dengue-Alert-Feb17-2011.pdf.

[5] Martin SanJL, Brathwaite O, Zambrano B, Solorzano JO, Bouckenooghe A, Dayan GH (2010). The epidemiology of dengue in the Americas over the last three decades: a worrisome reality.. Am J Trop Med Hyg..

[6] Teixeira MG, Costa MC, Coelho G, Barreto ML (2008). Recent shift in age pattern of dengue hemorrhagic fever, Brazil.. Emerg Infect Dis..

[7] Pan American Health Organization Number of reported cases of dengue and figures for 2010 (to week noted by each country). Epidemiological Week / EW 49. [updated 10 December 2010; cited 18 December 2011]. Available from:. http://new.paho.org/hq/dmdocuments/2010/dengue_cases_2010_december_10_2%20.pdf.

[8] Pan American Health Organization Number of reported cases of dengue and figures for 2011 (to week noted by each country). Epidemiological Week / EW 49. [updated 9 December 2011; cited 18 December 2011]. Available from:. http://new.paho.org/hq/dmdocuments/2011/dengue_cases_2011_January_21_EW_3.pdf.

[9] Pan American Health Organization Factsheet: Dengue. 2011. [updated 19 August 2011; cited 18 December 2011]. Available from:. http://new.paho.org/hq/index.php?option=com_content&task=view&id=4493&Itemid=2479&lang=en.

[10] Pan American Health Organization Re-emergence of dengue in the Americas. Epidemiol Bull. 1997;18(2). Available from:. http://www.paho.org/english/sha/epibul_95-98/be972ree.htm#cau.

[11] Alirol E, Getaz L, Stoll B, Chappuis F, Loutan L (2011). Urbanisation and infectious diseases in a globalised world.. Lancet Infect Dis..

[12] Gomez-Dantes H, Willoquet JR (2009). Dengue in the Americas: challenges for prevention and control.. Cad Saude Publica.

[13] Barclay E (2008). Is climate change affecting dengue in the Americas?. Lancet..

[14] Cellule Interrégionale d'Épidémiologie Antilles Guyane. Integrated management strategy for dengue prevention and control in the Caribbean subregion. Bulletin de veille sanitaire (BVS). 2009;8. Available from:. http://www.invs.sante.fr/publications/bvs/antilles_guyane/2009/bvs_ag_2009_08.pdf.

[15] Tapia-Conyer R, Mendéz-Galván J, Burciaga-Zúñ�iga P (2012). Community participation in the prevention and control of dengue: the patio limpio strategy in Mexico. Paediatr Int Child Health..

[16] Tapia-Conyer R, Mendez-Galvan JF, Gallardo-Rincon H (2009). The growing burden of dengue in Latin America.. J Clin Virol.

[17] Guy B, Barrere B, Malinowski C, Teyssou R, Lang J (2011). From research to Phase III: Preclinical, industrial and clinical development of the Sanofi Pasteur tetravalent dengue vaccine.. Vaccine..

